# Supplementary material for: Ensemble of nucleic acid absolute quantitation modules for copy number variation detection and RNA profiling
Source: Nat Commun. 2022 Apr 4;13:1791. doi: 10.1038/s41467-022-29487-y (PMC8979981; doi:10.1038/s41467-022-29487-y)
Supplement: Supplementary file 1 — Supplementary Information [file 41467_2022_29487_MOESM1_ESM.pdf]

Supplementary Information for

Ensemble of Nucleic Acid Absolute Quantitation Modules for Copy Number Variation Detection and RNA Profiling

Lucia Ruojia Wu[1,2#], Peng Dai[1,3#], Michael Xiangjiang Wang[1], Sherry Xi Chen[1,3], Evan N. Cohen[4], Gitanjali Jayachandran[4], Jinny Xuemeng Zhang[3], Angela V. Serrano[3], Nina Guanyi Xie[1], Naoto T. Ueno[5], James M. Reuben[4], Carlos H. Barcenas[5*], David Yu Zhang[3*]

[1] Department of Bioengineering, Rice University, Houston, TX, USA.

[2] School of Pharmaceutical Sciences, Capital Medical University, Beijing, China

[3]NuProbe USA, Houston, TX, USA.

[4]Department of Hematopathology, The University of Texas MD Anderson Cancer Center, Houston, TX, USA

[5]Department of Breast Medical Oncology, The University of Texas MD Anderson Cancer Center, Houston, TX, USA

[#]These authors contributed equally: Lucia Ruojia Wu, Peng Dai

*Correspondence to: genomic.dave@gmail.com

CHBarcenas@mdanderson.org

Table of Contents

Supplementary Note 1. Challenges in CNV detection .... Page 2
Supplementary Note 2. Two-plex QASeq .... Page 3
Supplementary Note 3. Multiplexed QASeq with >2 quantitation modules .... Page 5
Supplementary Note 4. Mutation analysis by QASeq .... Page 11
Supplementary Note 5. RNA expression level analysis by QASeq .... Page 13
Supplementary Note 6. Supplementary Methods and Discussions...................................... Page 20
Supplementary Note 7. Patient Characteristics.. Page 29

Supplementary Note 1. Challenges in CNV detection

Stochasticity in molecule sampling process is a significant challenge for detecting CNV. It leads to the observed number of DNA molecules, and thus the observed ploidy, deviating from the expected "true value" in CNV quantitation.

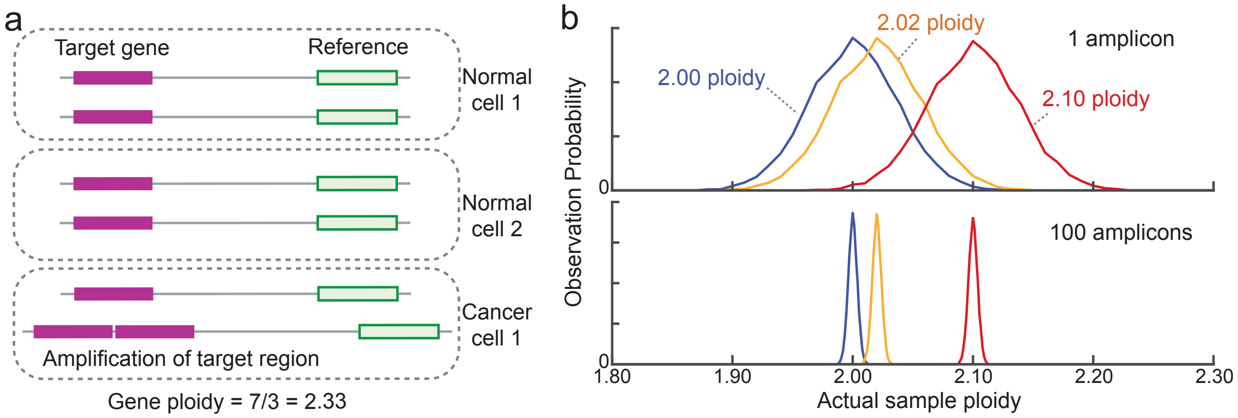

Figure S1. Challenges for detecting DNA copy number variations (CNVs). (a) Illustration of gene ploidy in a sample. Cancer cells are often mixed with normal cells in a sample, resulting in non-integer ploidy numbers. As the tumor load of a sample decreases, the ploidy approaches 2 and becomes difficult to detect by NGS or other methods. (b) Limits on CNV detection due to Poisson sampling error. The number of DNA molecules in a sample at a particular genetic locus follows a Poisson distribution. The top panel shows the distribution of actual ploidy of a gene in a sample, given different tumor ploidy values. The overlapping distributions indicate that it is not possible to confidently call even 2.10 ploidy. The Poisson distribution limitation can be overcome through increasing the number of independent genetic loci observed. The bottom panel shows the distribution of actual ploidy in a sample when considering the sum of 100 different amplicons.

Supplementary Note 2. Two-plex QASeq

NGS data alignment.

Each read in the original paired-end sequencing data consists of four sequences (from left to right): spacer, UMI, amplicon and adapter. Since the longest amplicon has 112 nt and the lengths of spacer and UMI are 4 nt and 15 nt respectively, their total length is shorter than the sequencing read length of 150 nt, and adapter sequence would appear at the end of each read. In order to align each read to the reference properly, the adapter "tails" are trimmed.

Adapter trimming is performed with following steps: a) for each forward read, spacer and UMI are trimmed off and UMI of each read is recorded; b) the overlapping part of the forward and reverse read is compared; if they are not perfectly matched, then both reads are abandoned; c) if the overlapping part is longer than 41 nucleotides, then it is written into a new fastq file for alignment. The purpose of step c is to filter out those reads that come from primer dimers as they are always shorter than on-target amplicons (the shortest amplicon is 50 nt).

Alignment is performed with an existing software Bowtie2 (version 2.4.1) with default parameters. The reference that the reads are aligned to is built from the amplicon sequences instead of the whole genome.

Calculation of UMI family count.

For each amplicon sequence, the aligned reads were grouped by UMI sequences; the group of reads carrying the same UMI sequence is called a "UMI family". The number of unique UMI families under one amplicon sequence is the "UMI family count", and the number of reads in each UMI family is the "UMI family size". We first removed UMI families containing obvious PCR errors, i.e. G bases found in the poly(H) UMI sequence). Next, we removed small UMI families which are likely the result of PCR-induced mutation or sequencing error in the UMI region. The cutoff for small UMI families was calculated as 5% of the mean of top 3 UMI family size values; all UMI families with a UMI family size ≤cutoff was removed. The number of accepted UMI families was the UMI family count value used in next data analysis steps. Because each DNA strand is attached with a different UMI, each original DNA input molecule should generate to 2 UMI counts assuming perfect conversion yield.

Comparison between 2-plex QASeq and ddPCR

ddPCR CNV analysis is performed following Bio-Rad Droplet Digital PCR Applications Guide. ERBB2 copy number (ploidy) is determined by calculating the ratio of target species molecule concentration to the reference molecule concentration, times the number of copies of reference species in the genome (2 for reference EIF2C1 in this study):

$$ERBB2\ Ploidy = ERBB2\ concentration/EIF2C1\ concentration * 2$$

Copies per droplet of target species (ERBB2) and reference (EIF2C1) is calculated according to Poisson distribution:

$$Copies\ per\ droplet = -ln(1-p),$$

where p = fraction of positive droplets for ERBB2 or EIF2C1.

Spike-in cell-line DNA samples with different expected ERBB2 ploidy were assayed by 2-plex QASeq and ddPCR, and high correlation in calculated ploidy was observed between the methods:

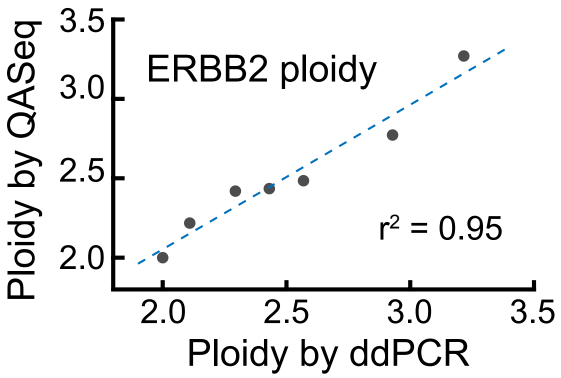

Figure S2. ERBB2 ploidy calculated from 2-plex QASeq and ddPCR. Spike-in samples with different expected ERBB2 ploidy were prepared by mixing a normal PBMC DNA sample and HER2-positive cell line (SK-BR-3) DNA.

Supplementary Note3. Multiplexed QASeq with >2 quantitation modules

Primer sequences for all QASeq panels are provided in Supplementary Data 1.

QASeq ploidy correlation with ddPCR in tumor samples.

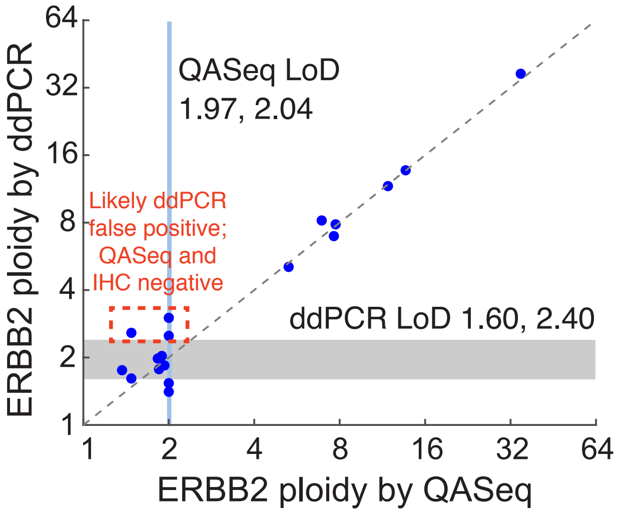

Figure S3. QASeq *ERBB2* ploidy quantitation is consistent with ddPCR in FF samples.

Pairwise analysis of ploidy using every 2 modules.

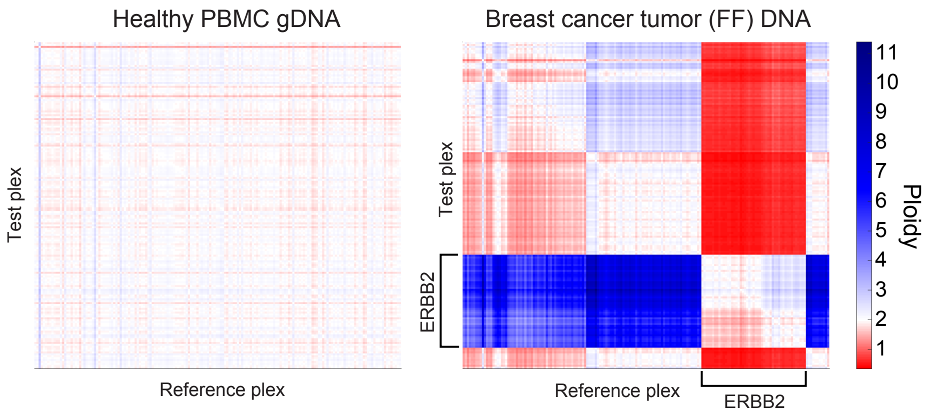

Figure S4. Pairwise ploidy analysis of 175-plex QASeq panel. Ploidy value can be calculated from the UMI family counts of any 2 modules as: ploidy = 2 × UMI family count of the test plex / UMI family count of the reference plex. In a healthy PBMC gDNA sample, the majority of ploidy values are close to 2. In a HER2-amplified FF DNA sample, the modules in ERBB2 can be distinguished from modules in other genomic regions.

Analysis of gene ploidy using >2 modules.

When analyzing QASeq panel with >2 quantitation modules, the first steps are NGS data alignment and calculation of UMI family count, which have been described in Supplementary Note2.

A simple way to calculate the ploidy of a gene with >2 modules is to use the ratio between the mean of UMI family counts in the gene of interest and in the reference regions. In

manuscript Fig. 2ab, ploidy of *ERBB2* is calculated as: ploidy = 2 × mean UMI family count in *ERBB2* / mean UMI family count in the reference. The ploidy for test sample was normalized against normal samples, with the mean *ERBB2* ploidy of normal samples set at 2.00. In the 175-plex QASeq panel, 49-plex are in ERBB2, 123-plex in other genomic regions are used as the reference. The rest 3-plex in Chromosome X are not used in CNV analysis; they are used for mutation calling only.

Data analysis of clinical samples.

Because cancer clinical samples often contain multiple CNV regions in the genome, using the mean of UMI family counts for calculation of ploidy may generate false positives. Therefore, we developed a workflow for robust CNV calling in complex clinical samples.

We first obtained the accurate ploidy for every amplicon in the 175-plex QASeq panel by calibrating the conversion yield χ of each plex using standard (healthy) samples and normalizing the UMI family counts in patient samples by χ. In this work, we tested 2 different types of samples: FF and cfDNA. We characterized several healthy samples for each different sample type: 10 healthy blood gDNA samples as the standard for FF patient samples, and 10 healthy blood plasma cfDNA samples for cfDNA patient samples. 2 healthy samples with the highest within-sample variability were excluded and not used for χ calculation.

After obtaining χ of healthy samples, we performed the following normalization for each amplicon in the panel in every patient sample: $normalized\ UMI\ family\ count = \frac{observed\ UMI\ family\ count}{\chi\ of\ the\ amplicon}$. Ploidy of each amplicon can be estimated as: $ploidy = \frac{normalized\ UMI\ family\ count}{sample\ input\ molecule\ number}$. The χ were calculated from the mean observed UMI family count of all healthy samples of the corresponding sample type.

Group name	Chromosome	Group size
ERBB4	2	14
PIK3CA	3	7
ESR1	6	3
EGFR	7	6
BRAF	7	2
PTEN	10	10
KRAS	12	1
ERBB3	12	5
BRCA2	13	9
AKT1	14	1
TP53	17	6
ERBB2	17	49
BRCA1	17	11
Chr17p	17	54
Chr17	17	114

Table S1. CNV groups of the 175-plex QASeq panel.

The QASeq panel can be used for CNV analysis of many target regions in the genome, including genes, sub-chromosomal regions, and chromosomes. The amplicons in the panel were grouped by which target region they are located. In Table S1, we showed the target regions (i.e. group names) included in the 175-plex panel, and the number of amplicons in each group (i.e.

group size). Note that some smaller groups are part of a larger group: group “TP53” is part of group “Chr17p”; group “Chr17p”, “ERBB2”, and “BRCA1” are part of “Chr17”.

In cancer cells, there could be complex CNVs, aneuploidies, and structural variations in any of the chromosomes; it is difficult to find a fixed reference genomic region with stable copy number in every tumor sample. Here we use a flexible approach to determining the reference; the workflow of the algorithm is shown in Figure S4.

Because the normalized family count is proportional to ploidy of the amplicon, using ploidy or normalized family count are equivalent in the rank-based Mann-Whitney U test. Here we use normalized family count for Mann-Whitney U test for consistency.

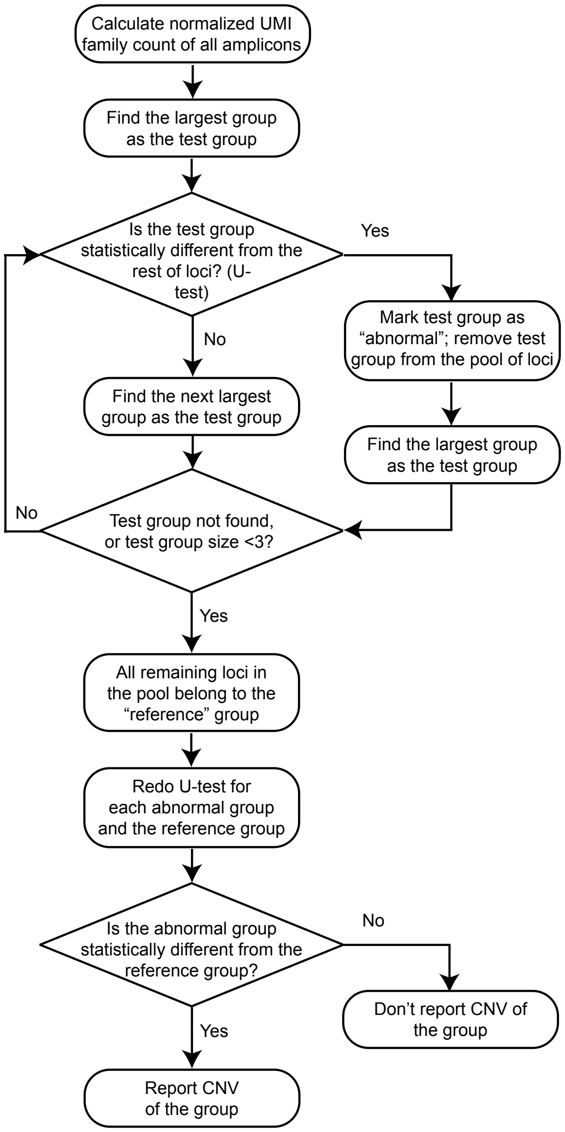

Figure S5. Workflow for copy number analysis, starting with normalized UMI family count.

We started with assigning the largest group as the test group, and all other amplicons in the panel as the reference group. A two-sided Mann-Whitney U test was performed to statistically test the null hypothesis that normalized UMI family count values in the test group have equal

median to normalized UMI family count values in the reference group, against the alternative hypothesis that they do not have equal median. If the null hypothesis is rejected statistically, this group will be marked as "abnormal group", and all amplicons from the current test group will be removed from the pool of amplicons and not to be included in later tests; the largest group in the remaining amplicons will be selected as the test group, and the rest of amplicons will become the reference group. If the null hypothesis cannot be rejected, the next largest group will become the test group, and the rest of amplicons (including the previous test group) will become the reference group. This process will be repeated until there is no group left, or the test group size is smaller than 3; the remaining amplicons in the pool will be the final reference group.

Note that everytime a group is marked as "abnormal", the next test group will be the largest group in the remaining amplicons, although it may have been tested in previous cycles; this is because the test needs to be redone when the reference is changed compared to previous cycles.

After determining the reference group, we redo Mann-Whitney U tests to see whether the "abnormal groups" are still statistically different from the final reference group. The "abnormal groups" with $p < \alpha$ will be reported for CNV; the ploidy of the group will be calculated as: ploidy = 2 × median normalized UMI family count in the current group / median normalized UMI family count in the reference group. For the groups with $p \geq \alpha$ and the groups not marked as "abnormal", we will report the ploidy as 2.00. Note that it is possible that CNV was detected for a smaller group within a larger group, but the larger group itself does not have CNV.

In manuscript Fig. 2f, the exact ploidy of each amplicon was calculated as: ploidy of the amplicon = 2 × normalized UMI family count of the amplicon / median normalized UMI family count in the reference group.

The Matlab function "ranksum()" was used for Mann-Whitney U test. We did not use groups with < 3 group size as the test group; these amplicons were included to analyze clinically relevant hotspot mutations and serve as reference in CNV analysis. Therefore BRAF, KRAS, and AKT1 are not included in the CNV status report; the amplicons in these 3 groups are included in the reference group.

The α value was adjusted based on Bonferroni correction; because 12 different groups were tested, there are 12 true null hypotheses, and α was adjusted as α = 0.05/12 = 0.0042.

Calculation of LoD.

In order to calculate the technical LoD of CNV detection, we tested aliquots of the same healthy blood gDNA sample 5 times using the 175-plex QASeq panel. For each experimental replicate, we calculated normalized UMI family count of each amplicon in the panel. The normalized UMI family count was calculated as: normalized UMI family count = observed UMI family count / standard χ; here the standard χ was the average χ of the other 4 experimental replicates.

Next, for each of the 49 amplicons in the ERBB2 group, the normalized UMI family count was multiplied by a fold change factor k. This new ERBB2 dataset was compared to the reference group (i.e. all non-ERBB2 amplicons); Mann-Whitney U test was performed to analyze whether the two groups have equal medians. We tested a lot of different k values ranging between 1.001 and 101 using a Matlab code, and found the minimum k value that generates a positive CNV gain test result for ERBB2. This minimum k value multiplied by 2 is the ploidy gain LoD of ERBB2.

Similarly, we also tested a lot of different *k* values ranging between 0.999 and 0, and found the maximum *k* value that generates a positive CNV loss test result for ERBB2. This maximum *k* value multiplied by 2 is the ploidy loss LoD of ERBB2.

We calculated gain LoD and loss LoD for each of the 5 experimental replicates; the median gain LoD and loss LoD of the 5 replicates are reported in Table S2. Other target CNV regions can be similarly analyzed for LoD; the median gain LoD and loss LoD of the 5 replicates are also included.

Group name	Group size	Ploidy gain LoD	Ploidy loss LoD
ERBB4	14	2.09	1.94
PIK3CA	7	2.1	1.91
ESR1	3	2.22	1.79
EGFR	6	2.11	1.9
PTEN	10	2.08	1.92
ERBB3	5	2.11	1.88
BRCA2	9	2.08	1.91
TP53	6	2.14	1.87
ERBB2	**49**	**2.04**	**1.97**
BRCA1	11	2.07	1.93
Chr17p	54	2.04	1.97
Chr17	114	2.04	1.96

Table S2. CNV LoD of all groups in the 175-plex QASeq panel.

Quantitation of *ERBB2* in healthy blood DNA samples using 175-plex QASeq.

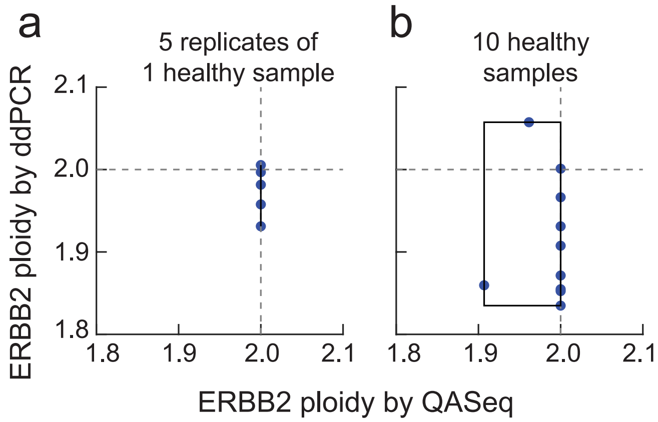

Figure S6. ERBB2 was quantitated using both ddPCR and 175-plex QASeq in healthy blood DNA samples. (a) The same healthy blood DNA sample was analyzed by both methods 5 times. ddPCR was performed using the ERBB2 copy number assay and EIF2C1 reference assay purchased from Bio-Rad; experimental and data analysis processes were performed according to Bio-Rad protocol. Using QASeq, we did not statistically observe CNV in ERBB2 by Mann-Whitney U test, so that the ploidy values are 2.00 for all 5 replicates. Using ddPCR, the ERBB2 ploidy ranges between 1.93 and 2.01. (b) 10 blood DNA samples from different healthy donors were analyzed using both methods; ddPCR showed wider ploidy range (1.84 to 2.06) than QASeq (1.91 to 2.00).

Summary of CNVs observed in tumor samples.

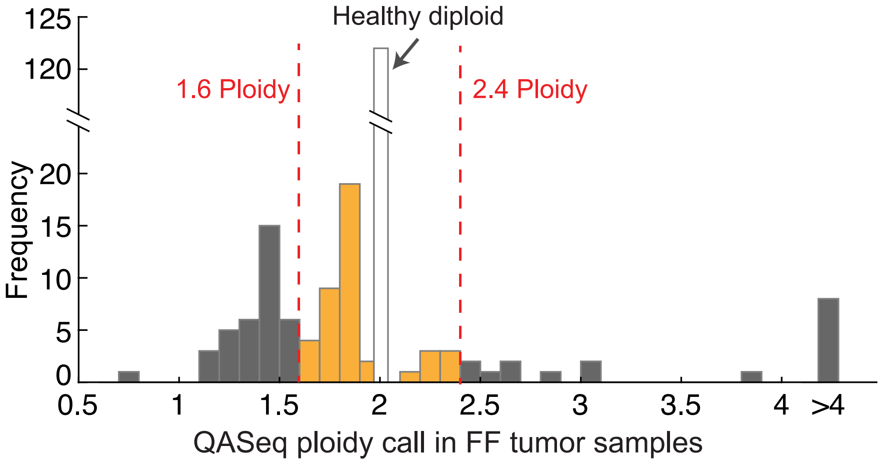

Figure S7. Histogram of observed gene ploidy values in 18 tumor DNA samples indicating improved clinical sensitivity.

Supplementary Note 4. Mutation analysis by QASeq

Primer sequences for QASeq panel (175 modules) are provided in Supplementary Data 1.

Mutation analysis workflow.

The first steps of NGS data processing for mutation analysis were the same as described in Supplementary Note S2, including the NGS reads alignment, grouping based on UMI sequence, removing UMI families containing PCR errors, and removing small UMI families which are likely the result of PCR-induced mutation in the UMI region.

We next determined the consensus sequences of the UMI families. In each amplicon sequence, the region between the 3′ of the forward primer and the 3′ of the inner reverse primer was used for mutation analysis; this region is called the identification region (IR). The IR sequence for each NGS read in the UMI family is identified. If any of the IR sequences in the UMI family are the same as the WT sequence (i.e. sequence from the human reference genome), the consensus sequence for this UMI family will be the WT sequence. Otherwise, a majority vote process is performed: if ≥70% reads of the UMI family contain the same IR sequence, this IR sequence will be the consensus sequence; if the most common IR sequence in the UMI family is present in <70% reads, this UMI family will be discarded. The number of UMI families of each different IR sequence is called the UMI family count.

Next, we compared the consensus sequences to the WT sequences and performed mutation calling. In order to reduce false positives, only mutations with UMI family count ≥ 3 and VAF ≥ 0.05% at the same time were further considered for variant calls. Here VAF of the mutation is calculated as VAF = UMI family count of the mutation / total UMI family count in the family.

In order to remove pseudogenes from the mutation list, we designed the primers to be different enough from the pseudogene sequences, so that the primer-pseudogene hybridization ΔG^o is weak in the PCR buffer, and the pseudogene templates cannot be amplified. There are still several pseudogene sequences that cannot be completely removed by primer design; we removed them during the process of mutation calling: if a consensus sequence contains "mutations" that appear in the pseudogene sequence, this UMI family will be removed.

Based on the initial test of the protocol on 10 healthy blood DNA samples and the Horizon WT reference sample, we observed some common false positives. If a mutation is observed in ≥3 healthy samples out of the 10 and has a VAF < 10%, it is unlikely a germline SNP; we think it is either a pseudogene that cannot be found by the online Basic Local Alignment Search Tool (BLAST), or an oligonucleotide contamination in the laboratory. This type of common false positives was removed from the mutation report. We also observed high frequency length change in homopolymer regions (i.e. ≥4 same consecutive bases); for example, AAAA>AAAAA and GGGGG>GGGG. It is known that polymerase and sequencing have higher indel rate in homopolymer regions than in normal regions; therefore, we did not report indel mutation in homopolymer regions.

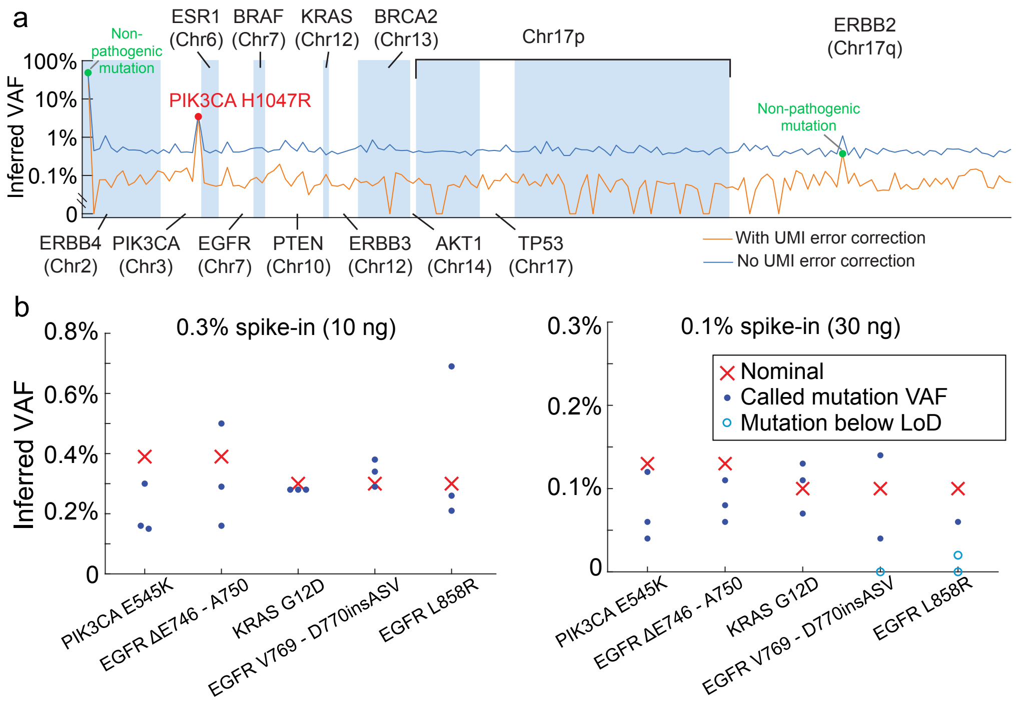

Figure S8. QASeq mutation analysis results. (a) Inferred mutation VAF with (orange line) and without (blue line) UMI correction for a breast cancer tumor section sample (8 ng input). Plotted here are the most dominant mutation in each amplicon; only mutations called are plotted as dots (red dots for pathogenic mutations, green dots for non-pathogenic mutations). The use of UMI bioinformatics in QASeq thus improves the mutation limit of detection by roughly 8-fold, from a background of roughly 0.8% to 0.1% VAF. (b) Validation of analytical sensitivity and specificity using Horizon reference cfDNA samples with 0.1% and 0.3% VAF mutations. At 10 ng DNA and 0.3% VAF, sensitivity was 100% (15/15). At 30 ng DNA and 0.1% VAF, sensitivity was 80% (12/15).

Supplementary Note 5. RNA expression level analysis by QASeq

QASeq Primer sequences for targeted RNA profiling panel are provided in Supplementary Data 1.

In QASeq, expression of each gene is calculated from the molecule count of each amplicon, and is further normalized relative to the reference genes. QASeq is compared with other technologies in this study.

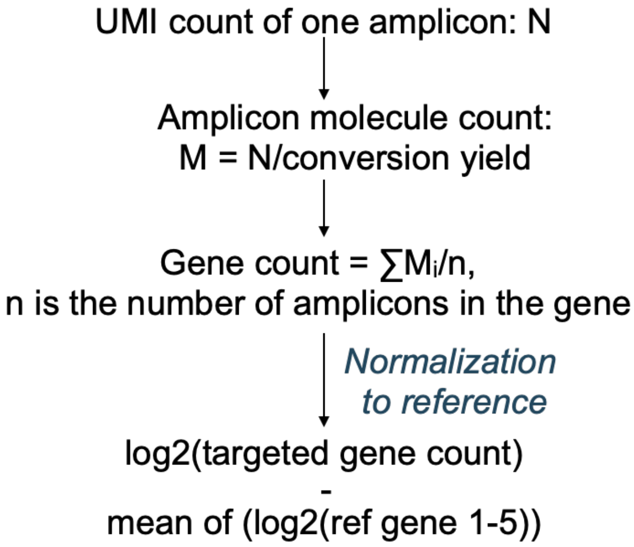

Figure S9. Expression level calculation formula.

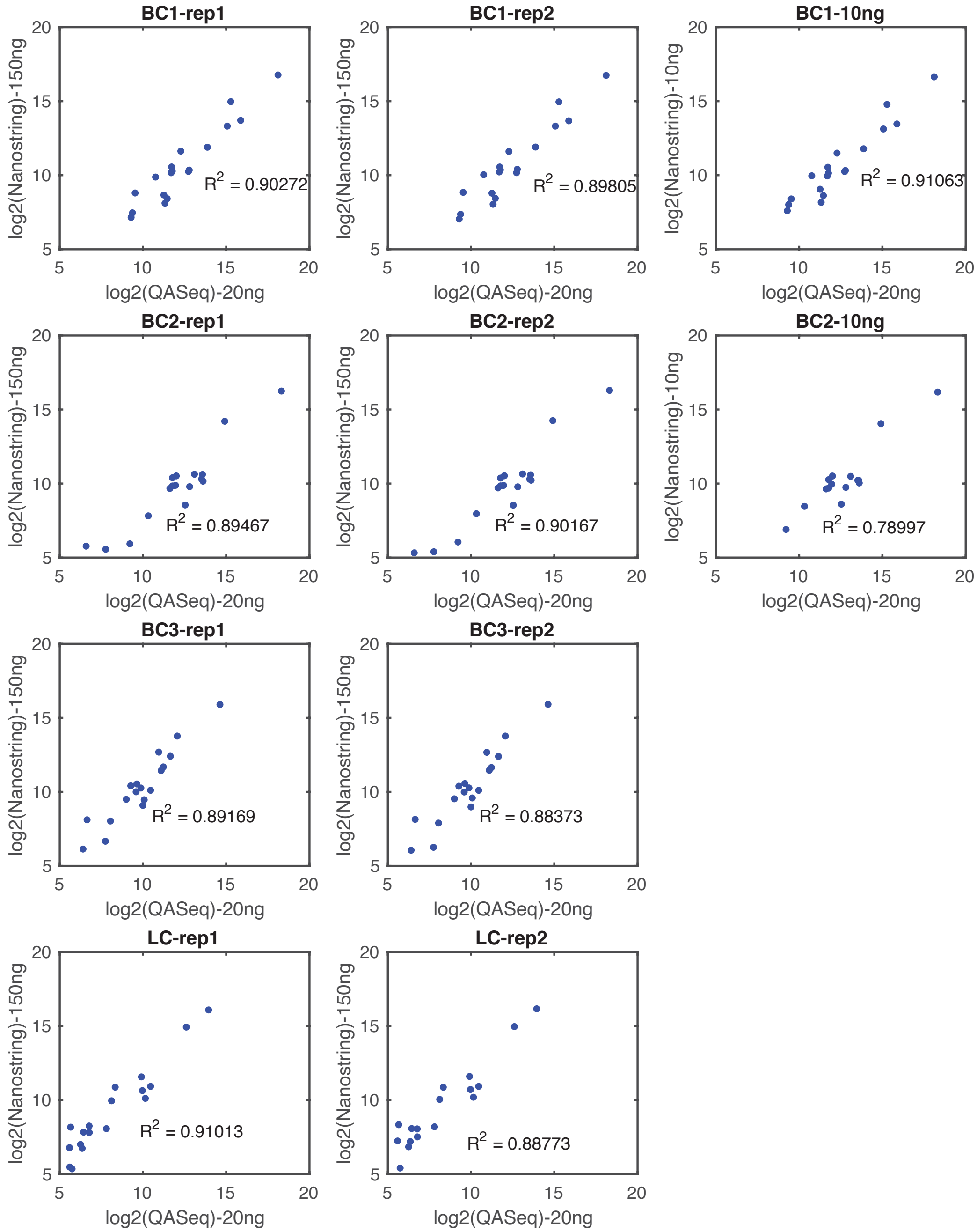

Figure S10. RNA expression level comparison between RNA QASeq and Nanostring.

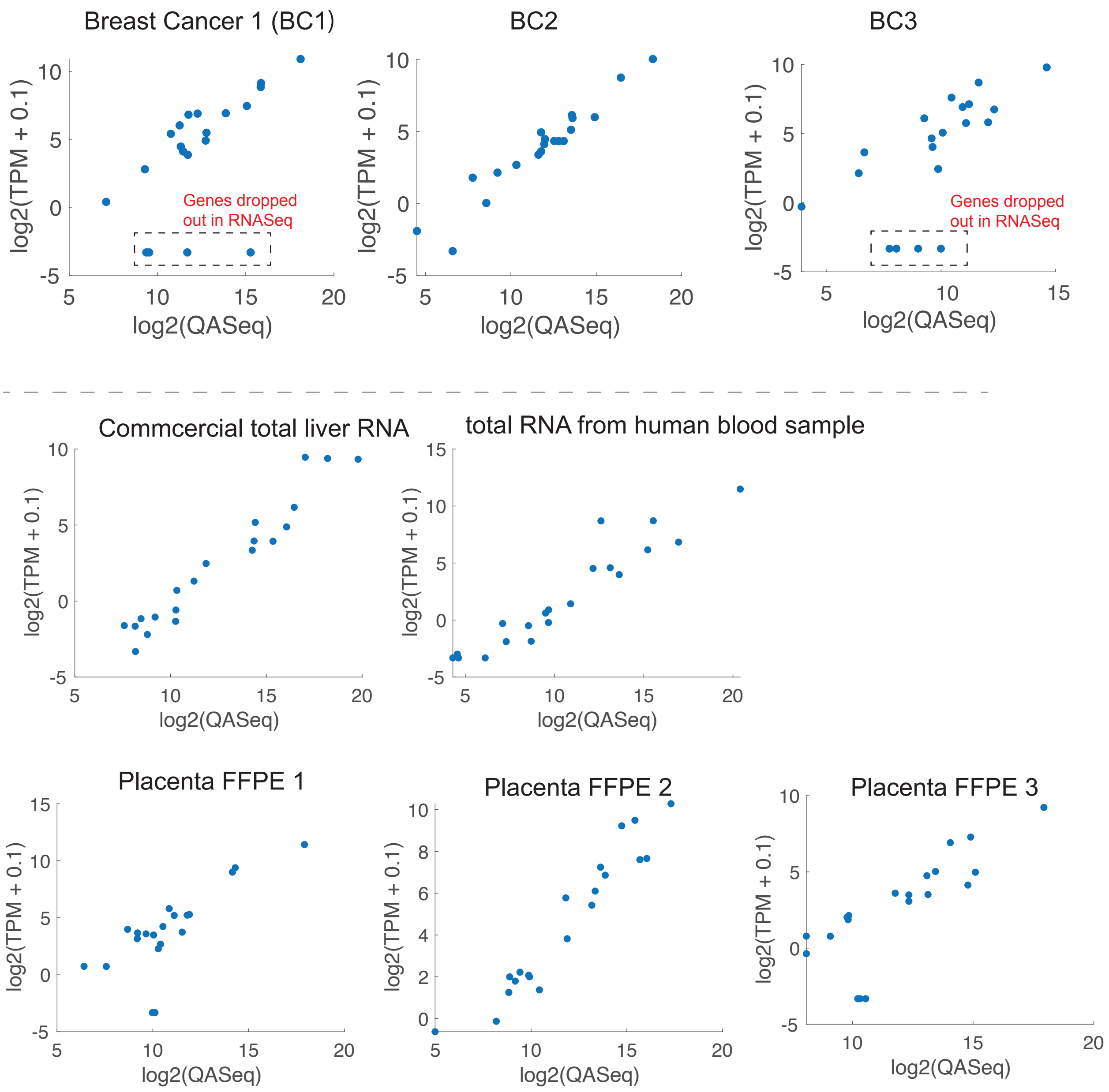

Figure S11. RNA expression level comparison between RNA QASeq and RNASeq. About 20 M reads are assigned for standard RNASeq, with ribosomal depletion. Low expression level gene may be dropped out in RNASeq especially in FFPE samples.

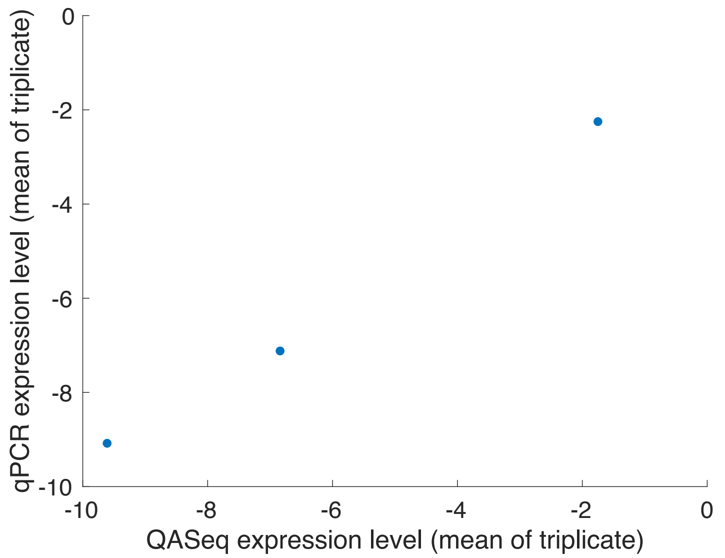

Figure S12. Comparison between QASeq and rt-qPCR. The expression level of three target genes (BAG1, MMP11, BIRC5) are normalized with five reference genes (TFRC, GUSB, RPLP0, ACTB, GAPDH). 10 ng human liver total RNA were used as input for each RNA QAseq library preparation or each rt-qPCR well. Both rt-qPCR and QASeq experiments were performed in triplicates and mean expression level are plotted. Linear regression $R^2 = 0.995$.

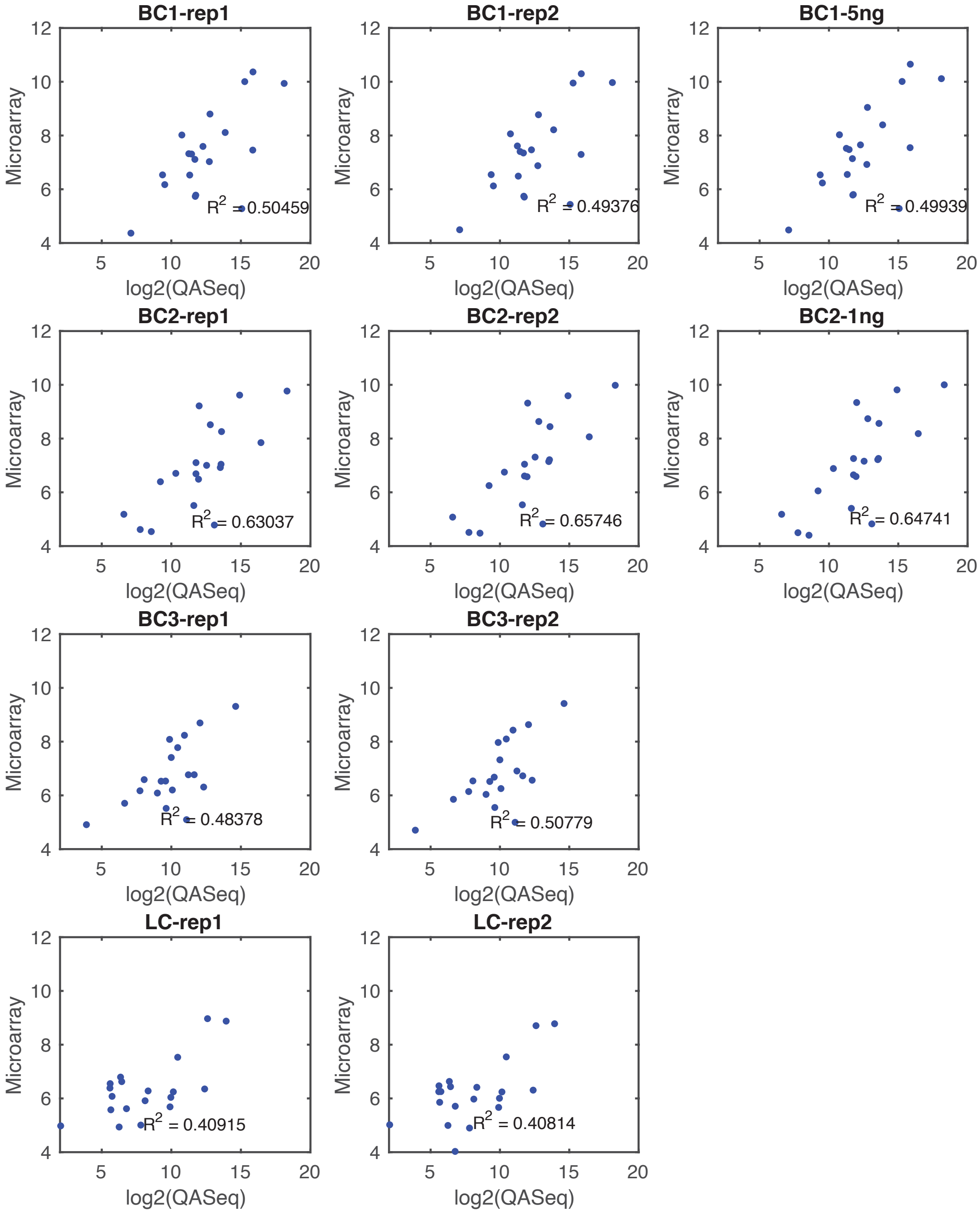

Figure S13. RNA expression level comparison between RNA QASeq and Microarray HTA.

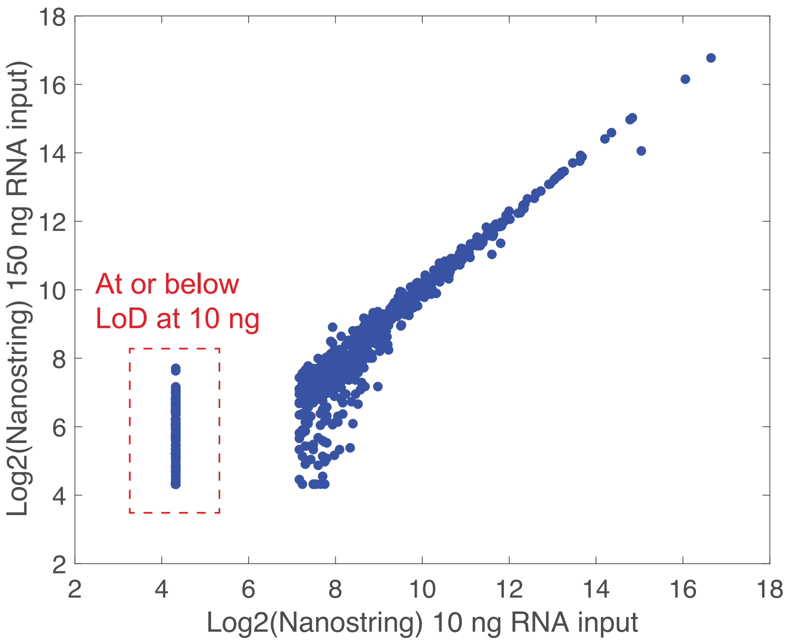

Figure S14. RNA expression level quantitation for the same FFPE RNA by Nanostring at different input amount.

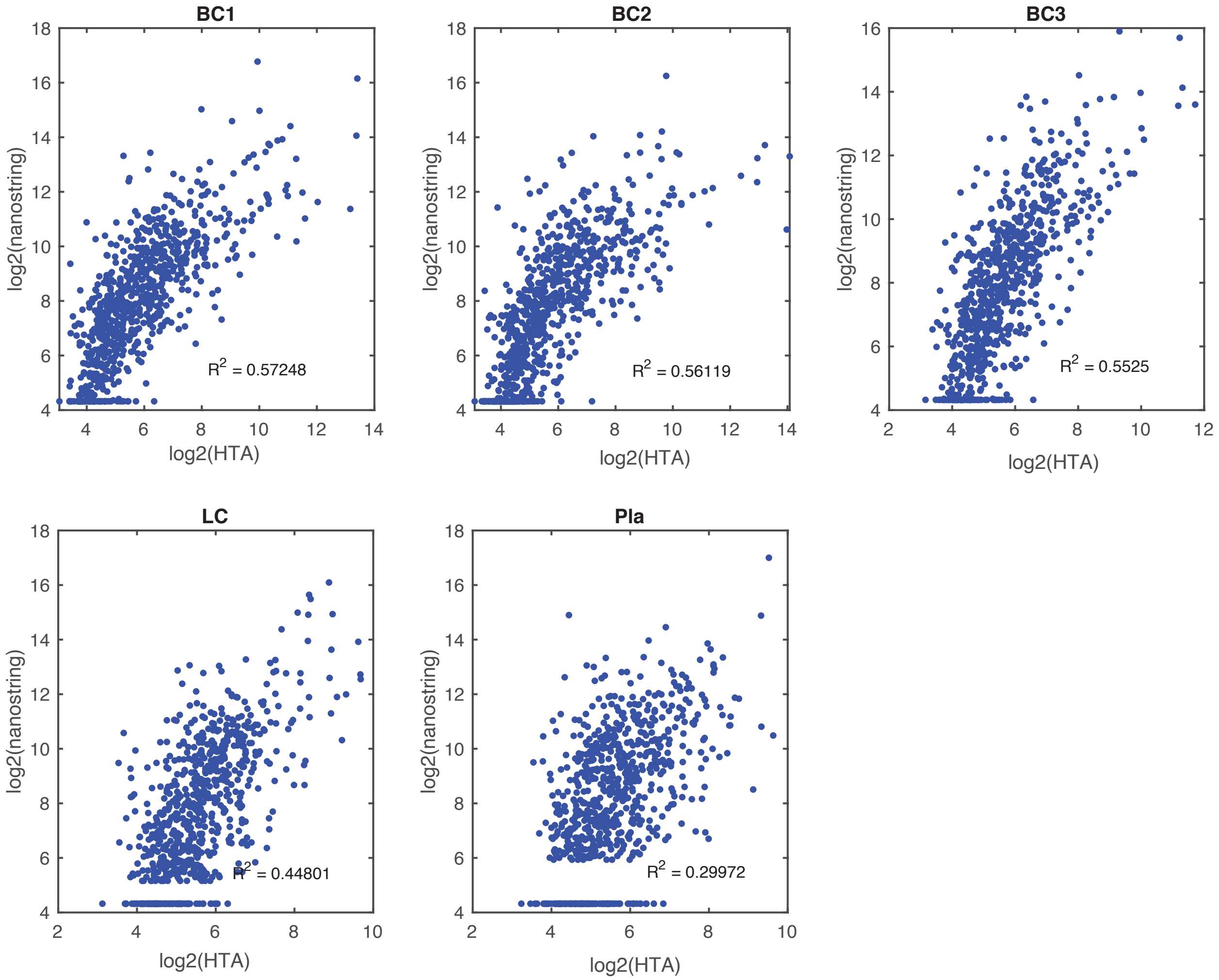

Figure S15. RNA expression level comparison between Nanostring and Microarray HTA.

Supplementary Note 6. Supplementary Methods and Notes
Recommended sequencing depth

The recommended sequencing depth is 90,000X with 8.3 ng human DNA input (approximately 5,000 haploid copies) in multiplexed QASeq. At 90,000X depth, 20 M reads is suggested for the 223-module QASeq breast cancer liquid biopsy panel when 8.3 ng input is used. The recommended sequencing depth should be adjusted proportionally with the input DNA amount, so that observed molecule count is not reduced due to insufficient reads.

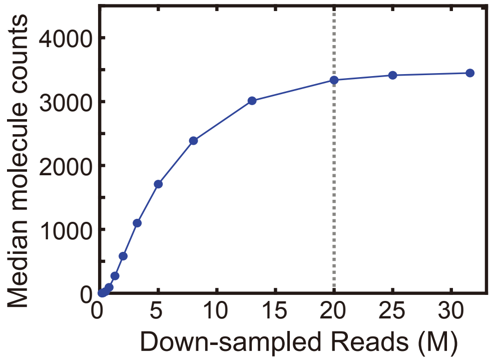

Figure S16. Observed molecule counts at different reads. From a 31.6M read FASTQ file for 175-plex QASeq panel at 10 ng gDNA input, the sequencing file was down-sampled to different subfile sizes. The median molecule counts for the 175 modules were summarized under different reads. The molecule counts firstly increase as more reads are assigned and then reach a stable plateau.

The relationship between observed molecule count and sequencing reads is illustrated in Figure S16. According to the recommendation of 90,000X depth with 8.3 ng DNA input, 108,000X depth should be used with 10 ng input, which corresponds to 19 M reads for 175-plex panel. This estimation is consistent with our observation that observed molecule counts reached plateau around 20 M reads.

Comparison of QASeq with other techniques

Table S3. Comparison of QASeq with WES and ddPCR

<table>
<tr><th></th><th>Cost</th><th>Sample preparation time</th><th>Quantitation Coverage</th><th>CNV LoD</th><th>Mutation LoD</th><th>Readout</th></tr>
<tr><td>QASeq</td><td>$30 -$250*</td><td>6 hours</td><td>1-223 regions</td><td>2.05 ploidy</td><td>0.1%</td><td rowspan="2">Sequencer</td></tr>
<tr><td>WES</td><td>~ $500</td><td>1-2 days</td><td>Whole exome (semi-quantitative)</td><td>~2.4 ploidy</td><td>2%</td></tr>
<tr><td>ddPCR</td><td>~ $30</td><td>4 hours</td><td>1-6 regions</td><td>~2.4 ploidy</td><td>0.1% or lower</td><td>Droplet Reader</td></tr>
</table>

*QASeq cost varies based on the number of quantitation modules in a panel and varies using different sequencing instrument. 20 M reads is suggested for the QASeq breast cancer liquid biopsy panel containing 223 modules. The sequencing cost using NextSeq 550 high output cartridge is about $200/sample. The sequencing cost will be significantly reduced to < $50/sample if Novaseq 6000 or Hiseq X system is used.

Low-plex QASeq DNA absolute quantitation modules showed comparable performance to ddPCR. With the scalability to highly multiplexed panels, QASeq improved CNV detection limit to below 2.05 ploidy. Furthermore, both CNV and mutation information are simultaneously provided from the NGS-based QASeq modules whereas ddPCR probes are designed for either CNV or mutation detection in one experiment.

Comparison of QASeq with CovCopCan, CNVKit and CODEX2

We compared QASeq with other CNV calling tools including CovCopCan and CNVKit for *ERBB2* copy number analysis in one normal PBMC DNA sample (expected *ERBB2* ploidy = 2.00), two reference spike-in samples prepared by mixing the normal PBMC DNA sample with *ERBB2*-positive cell line (SK-BR-3) DNA (expected *ERBB2* ploidy = 2.05 and 2.20), and three clinical cfDNA samples from breast cancer patients.

CovCopCan is designed for targeted sequencing, thus the analysis was performed using QASeq targeted sequencing data without considering UMI. Since CNVKit analysis is only compatible with whole exome sequencing (WES) or whole genome sequening (WGS), the six selected samples were also sent for WES at Yale Center for Genome Analysis (YCGA). CNVKit analysis was performed on the WES data with mean depth > 150X for all samples. As summarized in Table S4, Fig. 2b and Figure S17-S19, QASeq was able to distinguish spike-in reference samples and clinical cfDNA samples with ploidy ≥ 2.05 from the normal sample. CovCopCan and CNVkit were not able to detect *ERBB2* CNV in the 2.05 or 2.20 ploidy reference samples. CovCopCan, CNVkit and QASeq detected *ERBB2* amplification in 1, 2 and 3 samples out of the 3 clinical cfDNA DNA samples, respectively.

Based on these results, we believe QASeq has better CNV sensitivity than existing targeted sequencing-based or WES-based methods. Combining NGS-based accurate absolute quantitation module with high multiplexing to overcome molecule sampling stochasticity contributes to the improved CNV detection limit.

Table S4. Comparison of QASeq with CovCopCan and CNVkit.

<table>
<tr><td></td><td colspan="2">Targeted amplicon sequencing</td><td>Whole exome sequencing</td><td rowspan="2">Sample notes</td></tr>
<tr><td>Sample</td><td>**QASeq**</td><td>**CovCopCan**</td><td>**CNVkit**</td></tr>
<tr><td>Normal DNA</td><td>No CNV detected for *ERBB2*</td><td rowspan="5">No CNV detected for *ERBB2*</td><td rowspan="4">No CNV detected for *ERBB2*</td><td>Expected ploidy 2.00</td></tr>
<tr><td rowspan="2">Spike-in</td><td>2.06</td><td>Expected ploidy 2.05</td></tr>
<tr><td>2.28</td><td>Expected ploidy 2.20</td></tr>
<tr><td>Clinical cfDNA 3679</td><td>2.17</td><td>Case 3368 time point 2</td></tr>
<tr><td>Clinical cfDNA 3669</td><td>2.32</td><td>2.72 - 2.78</td><td>Case 3669 time point 1</td></tr>
<tr><td>Clinical cfDNA 3934</td><td>3.94</td><td>3.52</td><td>3.69 - 3.76</td><td>Case 3669 time point 2</td></tr>
</table>

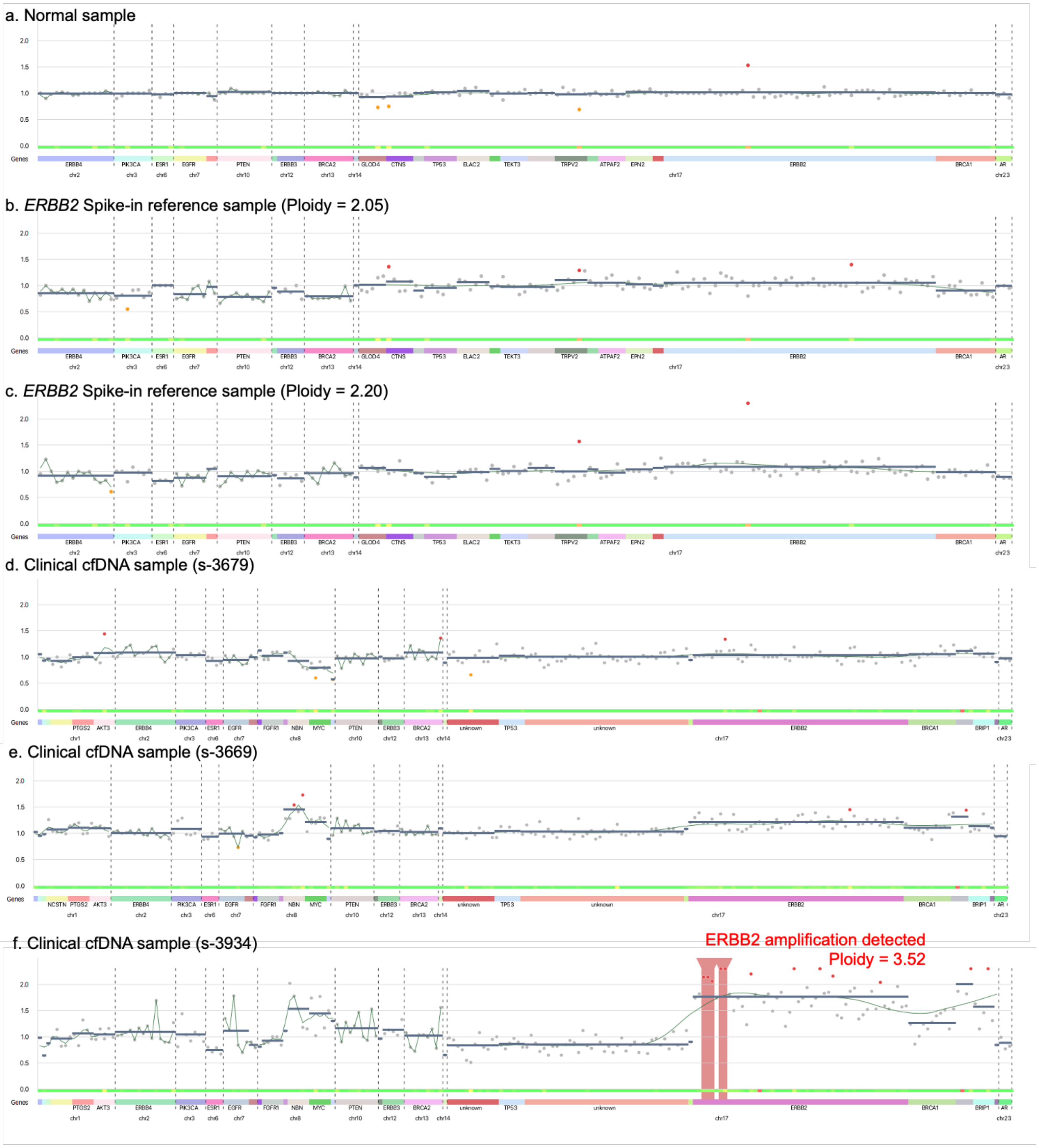

Figure S17. CovCopCan analysis of spike-in reference sample and clinical samples. Copy ratio plot was shown for normal sample (a), spike-in reference sample with expected ERBB2 ploidy of 2.05 (b), spike-in reference sample with expected ERBB2 ploidy of 2.20 (c), clinical sample S-3679 (d), clinical sample S-3669 (e), and clinical sample S-3934 (f).

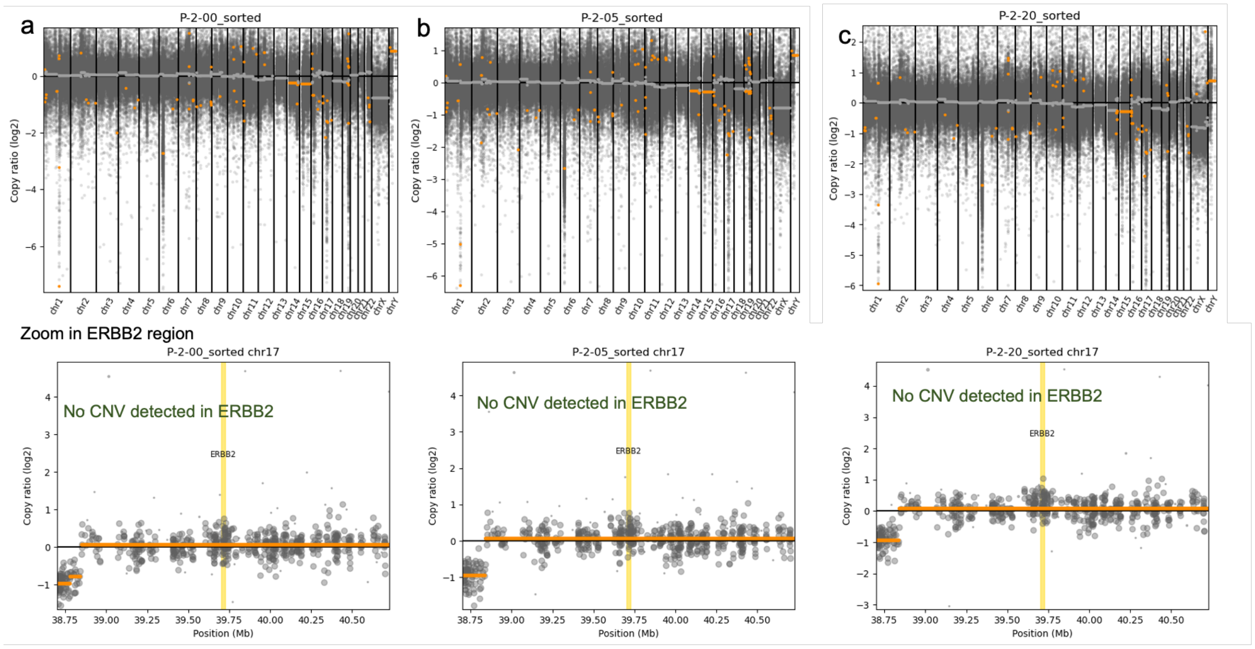

Figure S18. CNVKit analysis of spike-in sample WES data. Copy ratio scatter plot with zoom in for ERBB2 region was shown for normal sample (a), spike-in reference sample with expected ERBB2 ploidy of 2.05 (b), and spike-in reference sample with expected ERBB2 ploidy of 2.20 (c).

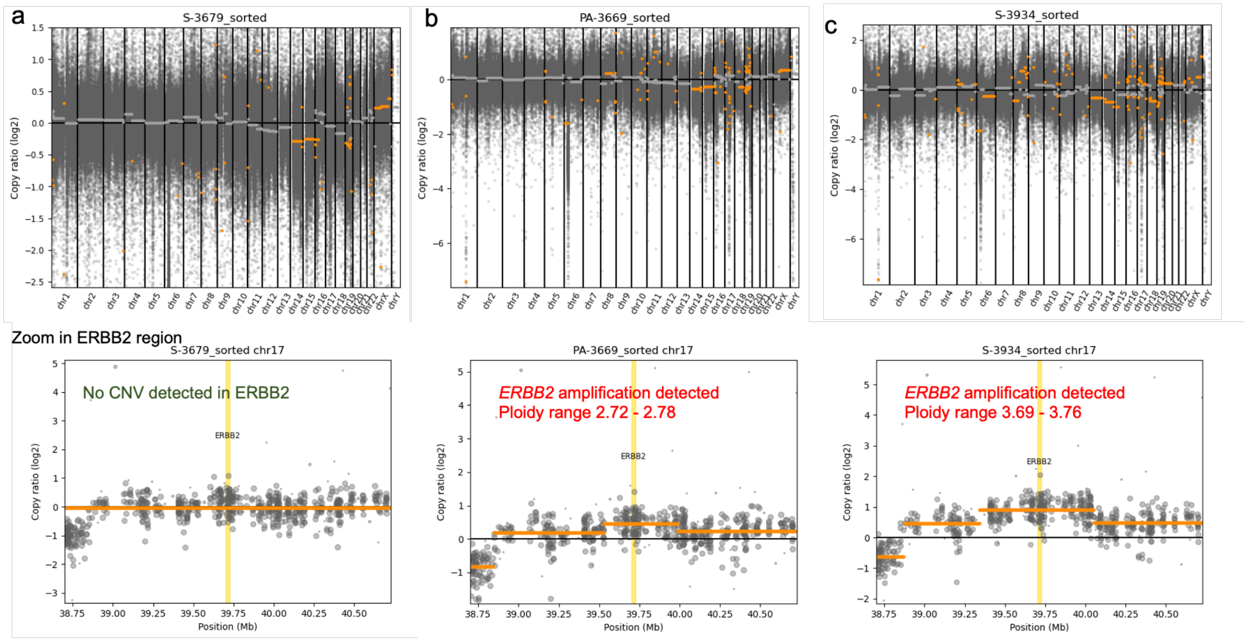

Figure S19. CNVKit analysis of clinical cfDNA sample WES data. Copy ratio scatter plot with zoom in for ERBB2 region was shown for clinical sample S-3679 (a), clinical sample S-3669 (b), and clinical sample S-3934 (c).

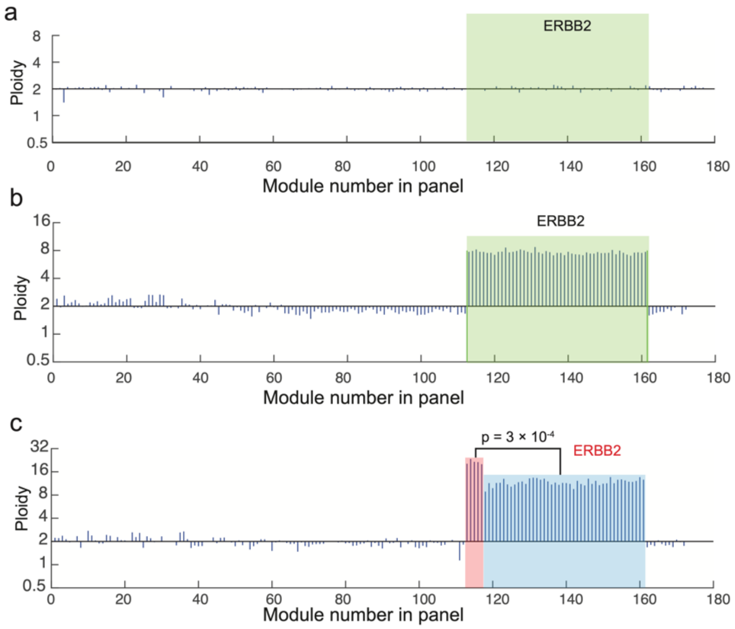

Figure S20. Representative performance of the different modules in the same target gene from (a) gDNA from a healthy donor PBMC; (b,c) gDNA from fresh/frozen tissue of two breast cancer patients. Modules in ERBB2 gene are highlighted. The ploidy values calculated from 49 different modules in ERBB2 are highly consistent as shown in (a) and (b). Moreover, sub-gene level copy number variation is detected in (c) since the first five modules in ERBB2 region are further amplified compared to the rest of ERBB2 modules. Here modules are sorted based on chromosome location.

QASeq panel design and scheme for library preparation workflow

The general design workflow consists of five steps:

1) Deciding the number of modules in gene of interest. The recommended number of modules in gene of interest is dependent on the desired limit of detection for copy number variation detection. As a reference for roughly estimating the number of modules for different LoD requirement, CNV LoD of different genes with different module numbers per gene in the 175-plex QASeq panel was summarized Supplementary Table S2 was summaried based on the performance of 175-plex QASeq panel and provided a reference for rough estimation of the number of modules for different LoD requirement.

2) Generate multiple primer candidates of forward primer (**fP**) and inner reverse primer (**rPin**) for each QASeq module. Genome context sequences based on regions of interest is downloaded. A single genome context sequence could have m fP candidates and n rPin candidates, thus combined into m×n primer pairs. Those primer pairs that satisfy specific amplicon length were selected as primer pair candidates for one module.

3) Optimize primer set of fP and rPin to minimize primer dimers for the whole panel, based on simulated annealing design using dimer likelihood estimation (SADDLE, Reference 31 in the manuscript), a primer set optimization software developed in our lab.

4) Based on the optimized fPs and rPins, generate multiple primer candidates for outer reverse primer (**rPout**). Candidates of rPout were generated so that the insert (the sequence between two primers) of fP and rPout was at least 4 nucleotides longer than the insert of fP and rPin for a nested design, to further reduce dimer or non-specific amplification.

5) Optimize primer set of fP and rPout to minimize primer dimers based on simulated annealing design using dimer likelihood estimation as previously mentioned.

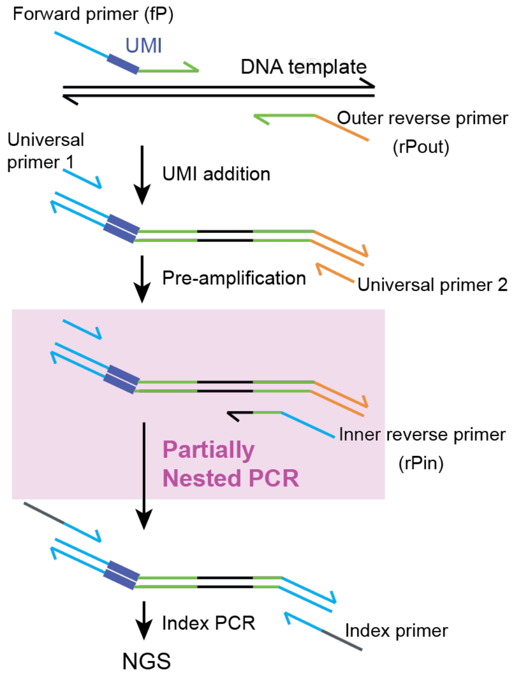

Figure S21. Scheme of QASeq library preparation workflow

UMI sequence

The degenerate base composition and length are optimized for QASeq panel. DNA sequences containing degenerate bases, such as poly(N) (i.e. mix of A, T, C, or G at each position), are often used as UMI sequences. In QASeq, we used **poly(H) (A, T, or C) as UMI**, because it has weaker cross-binding energy compared to poly(N) or mix of S (C or G) and W (A or T) bases as indicated by cross-binding energy calculation (Fig. S19).

The length of UMI determines how many molecules can be labeled uniquely. $\mathbf{H_{15}}$ contains 1.4×10^7 different sequences, which are enough for our planned molecule input. If 5,000 strands are used as input, H_{15} will allow 99.98% molecules to have unique UMI, and only 0.02% molecules may experience UMI collision by simulation. Even for 58,000 strands input (about 100 ng human gDNA), H_{15} will allow 99.6% molecules to have unique UMI.

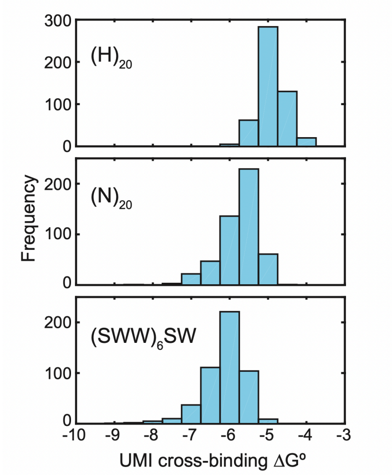

Figure S22. Simulation of UMI cross-binding energy. Using $(H)_{20}$ instead of $(N)_{20}$ or $(SWW)_6SW$ as UMI sequences reduces the mean cross-binding energy, indicating fewer potential primer-primer interactions to form dimers. Here 500 simulations were performed for each UMI pattern; in each simulation, 2 sequences that are consistent with the pattern were randomly generated, and the cross-binding ΔG° between these sequences were calculated assuming 60 °C and 0.18 M K^+.

Dynamic cutoff for copy number calculation.

UMI family size cutoff is essential for accurate and robust quantitation, because large number of UMI families with small UMI family size (< 3) were observed which could be results of polymerase and sequencing errors in the UMI sequence. Although we removed UMIs not matching the poly H (A,T,C, no G) UMI design, small families split from large families due to UMI mutations were not fully removed.

Different cutoffs were evaluated. The number of observed molecules will decrease as the family size cutoff increases. X% of the mean of top 3 largest family size were tested as the cutoff, where X = 0 (no cutoff), 3, 5, 10, 15, 20, 25 and 30. The calculated *ERBB2* ploidy using 2-plex panel was summarized in Figure S23.

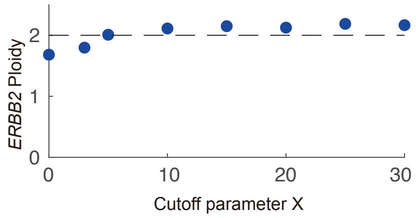

Figure S23. Calculated ERBB2 ploidy with different UMI family size cutoff. The cutoff was set as X% of the mean of top 3 largest family sizes. 2-plex QASeq panel as shown in manuscript Fig. 1b was analyzed here.

We showed that cutoff is necessary to get correct ploidy around 2 in a normal sample. There is no significant influence when the cutoff is larger than 5% of the mean of top 3. Furthermore, we evaluated the robustness in five technical replicates and selected X = 5 which minimized the variation (CV) of CNV quantitation in technical replicates.

Lowest tumor fraction for QASeq to detect

The tumor fraction that must be present in a sample for QASeq to call a CNV event is dependent on the ploidy of tumor tissue. With the ability to distinguish 2.05 ploidy from normal case, the lowest tumor fraction that can be detected is 0.5% assuming tumor gene ploidy is 12 (high amplification). The miminum tumor fraction will be reduced to 2.5% when tumor ploidy is 4.

The LoD for copy number loss is calculated to be 1.97 for ERBB2 gene in Table S2. Based on this LoD, the lowest tumor fraction that can be detected is 3% assuming tumor gene ploidy is 1, in the case of heterozygous single copy loss.

Supplementary Note 7. Patient Characteristics

Age	mean	52.7	
	median	55	
	min	28	
	max	75	
Race and/or Ethnicity	White	9	60%
	African American	3	20%
	Spanish/Hispanic	1	7%
	Asian/Pacifica Islander	1	7%
	Unknown	1	7%
Stage	IV	15	100%
Gender	Female	15	100%
Hormone Receptor and HER2 status, Primary	ER-	4	27%
	ER+	11	73%
	PR-	8	53%
	PR+	7	47%
	HER2+	15	100%
Hormone Receptor and HER2 status, Metastasis	ER-	4	27%
	ER+	11	73%
	PR-	9	60%
	PR+	6	40%
	HER2-	3	20%
	HER2+	12	80%
