# Supplementary material for: Ensemble of nucleic acid absolute quantitation modules for copy number variation detection and RNA profiling
Source: Nat Commun. 2022 Apr 4;13:1791. doi: 10.1038/s41467-022-29487-y (PMC8979981; doi:10.1038/s41467-022-29487-y)
Supplement: Supplementary file 3 — Description of Additional Supplementary Information [file 41467_2022_29487_MOESM3_ESM.pdf]

File name: Supplementary Data 1 QASeq primer sequences
Description: Primer sequences for QASeq panels
