# Supplementary material for: Ensemble of nucleic acid absolute quantitation modules for copy number variation detection and RNA profiling
Source: Nat Commun. 2022 Apr 4;13:1791. doi: 10.1038/s41467-022-29487-y (PMC8979981; doi:10.1038/s41467-022-29487-y)
Supplement: Supplementary file 5 — Reporting Summary [file 41467_2022_29487_MOESM5_ESM.pdf]

nature portfolio

Corresponding author(s): David Y Zhang

Last updated by author(s): Mar 7, 2022

Reporting Summary

Nature Portfolio wishes to improve the reproducibility of the work that we publish. This form provides structure for consistency and transparency in reporting. For further information on Nature Portfolio policies, see our Editorial Policies and the Editorial Policy Checklist.

Statistics

For all statistical analyses, confirm that the following items are present in the figure legend, table legend, main text, or Methods section.

n/a	Confirmed	
☐	☒	The exact sample size (*n*) for each experimental group/condition, given as a discrete number and unit of measurement
☐	☒	A statement on whether measurements were taken from distinct samples or whether the same sample was measured repeatedly
☐	☒	The statistical test(s) used AND whether they are one- or two-sided<br>*Only common tests should be described solely by name; describe more complex techniques in the Methods section.*
☐	☒	A description of all covariates tested
☐	☒	A description of any assumptions or corrections, such as tests of normality and adjustment for multiple comparisons
☒	☐	A full description of the statistical parameters including central tendency (e.g. means) or other basic estimates (e.g. regression coefficient) AND variation (e.g. standard deviation) or associated estimates of uncertainty (e.g. confidence intervals)
☐	☒	For null hypothesis testing, the test statistic (e.g. *F*, *t*, *r*) with confidence intervals, effect sizes, degrees of freedom and *P* value noted<br>*Give P values as exact values whenever suitable.*
☒	☐	For Bayesian analysis, information on the choice of priors and Markov chain Monte Carlo settings
☒	☐	For hierarchical and complex designs, identification of the appropriate level for tests and full reporting of outcomes
☒	☐	Estimates of effect sizes (e.g. Cohen's *d*, Pearson's *r*), indicating how they were calculated

Our web collection on statistics for biologists contains articles on many of the points above.

Software and code

Policy information about availability of computer code

Data collection	Sequencing data were collected using Illumina sequencers. Digital droplet PCR were performed using Bio-Rad QX200 Droplet Digital PCR System.
Data analysis	Code for QASeq NGS data analysis is available from Github (https://github.com/wrj915/QASeq). Raw fastq reads in RNAseq were initially quality filtered using Trimmomatic v0.39. Alignment was performed using the Bowtie2 software. Digital droplet PCR data were analyzed using Bio-Rad Quantasoft Software v1.4

For manuscripts utilizing custom algorithms or software that are central to the research but not yet described in published literature, software must be made available to editors and reviewers. We strongly encourage code deposition in a community repository (e.g. GitHub). See the Nature Portfolio guidelines for submitting code & software for further information.

Data

Policy information about availability of data

All manuscripts must include a data availability statement. This statement should provide the following information, where applicable:

- Accession codes, unique identifiers, or web links for publicly available datasets
- A description of any restrictions on data availability
- For clinical datasets or third party data, please ensure that the statement adheres to our policy

The main data supporting the results in this study are available within the paper and its Supplementary Information. The raw sequencing data and ddPCR data generated in this study have been deposited in NCBI Sequence Read Archive under BioProject ID PRJNA813699 (https://www.ncbi.nlm.nih.gov/bioproject/PRJNA813699) and Figshare https://doi.org/10.6084/m9.figshare.16529640. Human reference genome (GRCh38) used in the study for alignment is accessed from NCBI under BioProject ID PRJNA31257. Source data are provided with this paper.

Field-specific reporting

Please select the one below that is the best fit for your research. If you are not sure, read the appropriate sections before making your selection.

☒ Life sciences ☐ Behavioural & social sciences ☐ Ecological, evolutionary & environmental sciences

For a reference copy of the document with all sections, see nature.com/documents/nr-reporting-summary-flat.pdf

Life sciences study design

All studies must disclose on these points even when the disclosure is negative.

Sample size	No sample size calculation was carried out during experiment design. Clinical sample size was determined by availability.
Data exclusions	No sample was excluded.
Replication	The analytical performance of 2-plex QASeq assays were performed in five replicates; all attempts at replication were successful. The analytical performance of 2.00 ploidy sample was tested in 4 replicates and 2.05 ploidy sample was tested in duplicates; all attempts at replication were successful.
Randomization	Not relevant. Samples were obtained from the MD Anderson Cancer Center based on availability.
Blinding	For all clinically relevant studies the researchers were blinded to group allocation during data collection and analysis. Specifically, the clinical information of the breast cancer clinical samples including disease progression and tumor tissue FISH results were blinded to researchers performing QASeq NGS assay until the experiments and data analysis were complete. The researchers collecting the samples did not participate in QASeq assay or data analysis.

Reporting for specific materials, systems and methods

We require information from authors about some types of materials, experimental systems and methods used in many studies. Here, indicate whether each material, system or method listed is relevant to your study. If you are not sure if a list item applies to your research, read the appropriate section before selecting a response.

Materials & experimental systems

n/a	Involved in the study	
☒	☐	Antibodies
☒	☐	Eukaryotic cell lines
☒	☐	Palaeontology and archaeology
☒	☐	Animals and other organisms
☐	☒	Human research participants
☒	☐	Clinical data
☒	☐	Dual use research of concern

Methods

n/a	Involved in the study	
☒	☐	ChIP-seq
☒	☐	Flow cytometry
☒	☐	MRI-based neuroimaging

Human research participants

Policy information about studies involving human research participants

Population characteristics	All 15 patients had a diagnosis of ERBB2+ (IHC II+ or III+ or FISH+) metastatic breast cancer and the ERBB2 status was confirmed from a biopsy obtained from tumor tissue. Population characteristics including age, race/Ethnicity, stage, gender, hormone receptor and HER2 status are summarized in Supplementary Note 7 in the supplementary information.
Recruitment	All participants were recruited at MD Anderson Cancer Center. Blood samples were collected from patients diagnosed with metastatic breast cancer before starting the first line of systemic therapy for metastatic disease. Only patients with HER2-positive primary tumors were included in this study. Six de-identified plasma samples from 15 patients with HER2-positive metastatic breast cancer were collected at MD Anderson Cancer Center.
Ethics oversight	All procedures performed in studies involving human participants were approved by Institutional Review Board at MD Anderson (protocols PA16-0507 and PA19-0375), and were in accordance with the 1964 Helsinki declaration and its later amendments or comparable ethical standards. Informed consent was obtained from all participants who were not compensated, and the data was not used for any treatment decisions.

Note that full information on the approval of the study protocol must also be provided in the manuscript.
